# *MiR-202-5p* Regulates Geese Follicular Selection by Targeting *BTBD10* to Regulate Granulosa Cell Proliferation and Apoptosis

**DOI:** 10.3390/ijms24076792

**Published:** 2023-04-05

**Authors:** Mingxia Ran, Shenqiang Hu, Hengli Xie, Qingyuan Ouyang, Xi Zhang, Yueyue Lin, Xin Yuan, Jiwei Hu, Hua He, Hehe Liu, Liang Li, Jiwen Wang

**Affiliations:** Farm Animal Genetic Resources Exploration and Innovation Key Laboratory of Sichuan Province, Sichuan Agricultural University, Chengdu 611130, China; 18227585649@163.com (M.R.); sqhu2011@163.com (S.H.); xiehengli2022@163.com (H.X.); oyqy222@163.com (Q.O.); zhangx802802@163.com (X.Z.); lyy3078539326@163.com (Y.L.); hehua023@126.com (H.H.); cnliliang@foxmail.com (L.L.)

**Keywords:** follicular selection, granulosa cell, proliferation, apoptosis, *miR-202-5p*, *BTBD10*

## Abstract

The regulation of granulosa cells (GCs) proliferation and apoptosis is the key step in follicular selection which determines the egg production performance of poultry. *miR-202-5p* has been reported to be involved in regulating the proliferation and apoptosis of mammalian ovarian GCs. However, its role in regulating the proliferation and apoptosis of goose GCs is still unknown. In the present study, the GCs of pre-hierarchical follicles (phGCs, 8–10 mm) and those of hierarchical follicles (hGCs, F2–F4) were used to investigate the role of *miR-202-5p* in cell proliferation and apoptosis during follicle selection. In phGCs and hGCs cultured in vitro, *miR-202-5p* was found to negatively regulate cell proliferation and positively regulate cell apoptosis. The results of RNA-seq showed that BTB Domain Containing 10 (*BTBD10*) is predicted to be a key target gene for *miR-202-5p* to regulate the proliferation and apoptosis of GCs. Furthermore, it is confirmed that *miR-202-5p* can inhibit *BTBD10* expression by targeting its 3′UTR region, and *BTBD10* was revealed to promote the proliferation and inhibit the apoptosis of phGCs and hGCs. Additionally, co-transfection with *BTBD10* effectively prevented *miR-202-5p* mimic-induced cell apoptosis and the inhibition of cell proliferation. Meanwhile, *miR-202-5p* also remarkably inhibited the expression of Phosphatidylinositol-4,5-Bisphosphate 3-Kinase Catalytic Subunit Beta (*PIK3CB)* and AKT Serine/Threonine Kinase 1 (*AKT1),* while it was significantly restored by *BTBD10*. Overall, *miR-202-5p* suppresses the proliferation and promotes the apoptosis of GCs through the downregulation of *PIK3CB*/*AKT1* signaling by targeting *BTBD10* during follicular selection. Our study provides a theoretical reference for understanding the molecular mechanism of goose follicular selection, as well as a candidate gene for molecular marker-assisted breeding to improve the geese’ egg production performance.

## 1. Introduction

Compared to chickens and ducks, which typically have an annual egg production of about 200 to 300 eggs, geese only produce, on average, around 100 eggs per year. Furthermore, most breeds of geese only produce between 20 and 40 eggs annually, which greatly limits the potential for growth and development in the goose industry. The egg production performance of avian species is dependent upon the maintenance of follicular selection, a process in which a single follicle is selected to initiate rapid growth and final differentiation before ovulation [1]. During follicle selection, more than 90% of the follicles are not selected and, eventually, become atretic [2,3]. As the main component of the follicle wall, accumulating evidence indicates that follicular atresia is thought to be mainly caused by apoptosis of granulosa cells (GCs) [3,4,5,6,7]. Meanwhile, in hierarchical follicles the FSH will suppress GCs apoptosis which prevent follicles from atresia. Then, as the expression of luteinizing hormone receptor (LHR) increases, LH will prevent preovulatory follicle atresia by further inhibiting granulosa cell apoptosis [8,9,10]. These studies indicate that the regulation of GCs proliferation and apoptosis is the key step of follicular selection. Therefore, understanding the molecular mechanism that regulates the GCs proliferation and apoptosis during goose follicle selection can provide an important theoretical reference for improving goose egg production.

MicroRNAs (miRNAs) are endogenous non-coding RNAs that can cause mRNA degradation or translation inhibition via imperfectly binding to their target mRNAs at their 3′ untranslated regions (3′UTRs) [11]. It is worth mentioning that miRNAs play significant roles in regulatory mechanisms operating in various bioprocesses, including follicular selection, by affecting the morphology and function of granulosa cells (such as proliferation and apoptosis) [12,13,14]. *miR-202-5p* was highly expressed in goose GCs, and exhibited a stage-dependent expression pattern in GCs in different-sized goose follicles [15], suggesting that it may have an important role in geese follicular selection. *miR-202-5p* is the most abundant mature miRNA of gonads in several vertebrate species [16,17,18]. Moreover, the expression of *miR-202-5p* is also closely related to oogenesis [18,19]. These results reveal the important regulatory role of *miR-202-5p* in female reproduction; however, its specific impact and regulatory mechanism in geese’ follicular selection remained elusive.

To address the role of *miR-202-5p* in geese follicular selection, in the present study, we investigate the influence of overexpression/interference of *miR-202-5p* on the proliferation and apoptosis of pre-hierarchical follicles and hierarchical follicles GCs. Additionally, we conducted RNA-seq to explore its regulatory mechanism and transcriptional target. These results are expected to provide valuable theoretical references for understanding the molecular mechanism of goose follicular selection.

## 2. Results

### 2.1. Identification of miR-202-5p Expression Pattern

**Figure 1:**
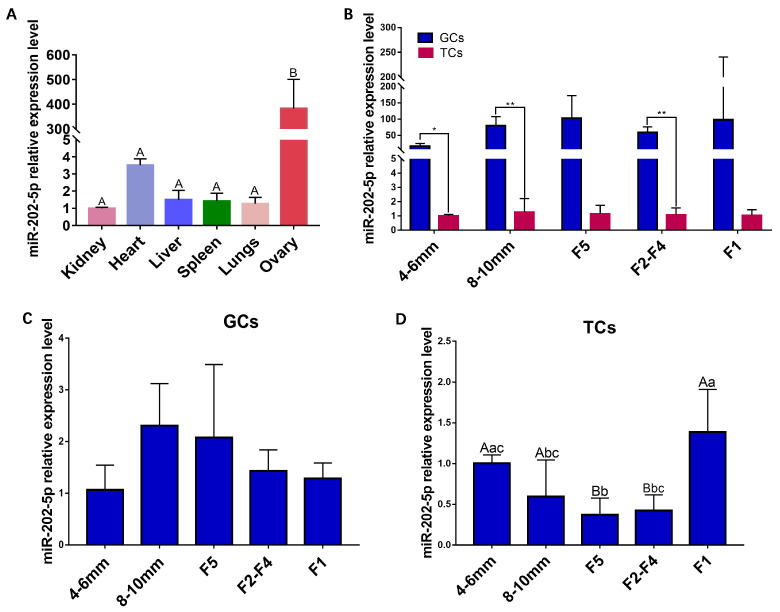
Tissue distribution pattern of *miR-202-5p* by RT–PCR. (**A**): Relative expression of *miR-202-5p* in different visceral tissues (n = 3). (**B**): The relative expression level of *miR-202-5p* in GCs compared to TCs (n = 3); Expression of *miR-202-5p* in GCs (**C**) or TCs (**D**) of follicles at different stages (n = 3). Data are means ± SD of technical replicates. There is no significant difference if there is the same mark letter, and there is a significant difference if there are different mark letters. Lowercase letters represent *p* < 0.05, and uppercase letters represent *p* < 0.01. “**” indicated a very significant difference among different groups (*p* < 0.01), “*” indicated a significant difference among different groups (*p* < 0.05).

The tissue distribution patterns of *miR-202-5p* in six tissues, including the heart, liver, spleen, lung, kidney, and ovary, were determined by quantitative reverse transcription polymerase chain reaction (RT-PCR). As shown in Figure 1A, compared to other tissues, *miR-202-5p* was much more prevalent in the ovaries (*p* < 0.01), indicating that *miR-202-5p* may be an important post-transcriptional regulator in regulating ovarian function. The follicle is the basic functional unit of avian ovarian ovulation [20]. A goose follicle consists mainly of oocytes and their surrounding somatic cells (GCs and theca cells (TCs)) [21]. Then, a further qRT-PCR was conducted to identify *miR-202-5p* expression levels in the GCs and TCs of goose follicles at different stages. It was found that *miR-202-5p* was significantly more expressed in GCs in the five stages of follicles than in TCs (Figure 1B) (*p* < 0.05). Furthermore, *miR-202-5p* showed a trend of increasing and then decreasing from pre-hierarchical follicles 4–6 mm, 8–10 mm, to hierarchical follicles F5, F2–4, and F1. However, there is no statistically significant difference between these changes (*p* > 0.05) (Figure 1C). In contrast, *miR-202-5p* expression in TCs showed a trend of first decreasing and then increasing from pre-hierarchical follicles 4–6 mm and 8–10 mm, to hierarchical follicles F5, F2–4, and F1, and, apart from the highly significant difference in changes observed between 8 and 10 mm pre-hierarchical follicles and F5 hierarchical follicles (*p* < 0.01), changes at other stages did not show a significant differences (*p* > 0.05) (Figure 1D). These results suggest that *miR-202-5p* may be involved in regulating the GCs during goose follicle selection.

### 2.2. miR-202-5p Suppresses GCs Proliferation and Induces GCs Apoptosis

**Figure 2:**
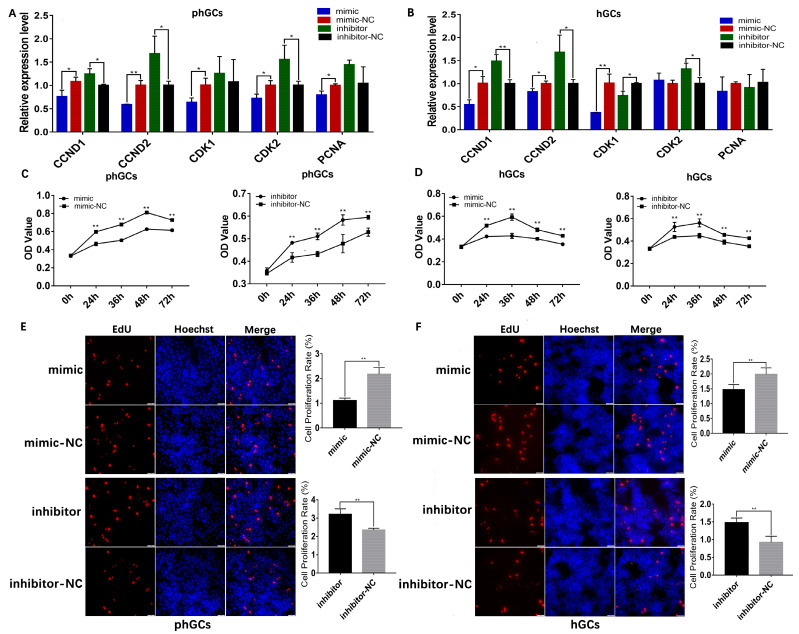
Effect of *miR-202-5p* overexpression/interference on the proliferation of phGCs and hGCs. Relative expression of proliferation-related genes in phGCs (**A**) and hGCs (**B**) (n = 3); the cell activity of phGCs (**C**) and hGCs (**D**) detected by CCK8 assay (n = 6); the changes in the proliferation rate of phGCs (**E**) and hGCs (**F**) detected by EdU staining (n = 3). Data are means ± SD of technical replicates. “**” indicated a very significant difference among different groups (*p* < 0.01), “*” indicated a significant difference among different groups (*p* < 0.05).

To investigate the biological functions of *miR-202-5p* in the proliferation of GCs, according to the expression profile of *miR-202-5p* in GCs of follicles at different stages, the pre-hierarchical follicles (phGCs, 8–10 mm) and those of hierarchical follicles (hGCs, F2–F4) were selected to in vitro transfected with *miR-202-5p* mimics or inhibitors. Subsequently, the result of qRT-PCR revealed that *miR-202-5p* mimic significantly inhibited, while inhibitor significantly promoted, the expression of cyclin D1 (*CCND1*), *CCND2* in phGCs (Figure 2A), and hGCs (Figure 2B). The effect of *miR-202-5p* on cell activity was further detected by CCK8 assay. As shown in Figure 1B,C, *miR-202-5p* mimic significantly inhibited the cell activity of phGCs and hGCs, whereas the inhibitor significantly promoted it (Figure 2C,D). Consistent with the results of qRT-PCR and CCK8, EdU staining results indicated that the mimic significantly decreased EdU-positive phGCs and hGCs, whereas the inhibitor significantly increased them (Figure 2E,F). These results all indicated that *miR-202-5p* is capable of inhibiting the proliferation of phGCs and hGCs.

**Figure 3:**
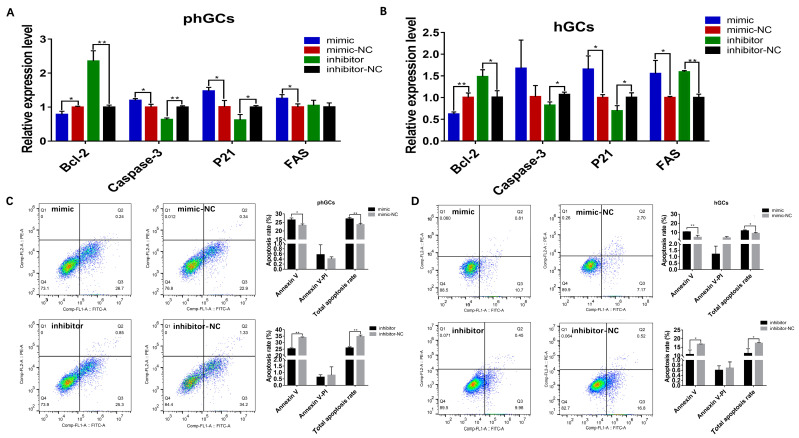
Effect of *miR-202-5p* overexpression/interference on the apoptosis of phGCs and hGCs. The relative expression of apoptosis–related genes in phGCs (**A**) and hGCs (**B**) (n = 3); apoptosis rates of phGCs (**C**) and hGCs (**D**) were determined by flow cytometer (n = 3). Data are means ± SD of technical replicates. * *p* < 0.05, ** *p* < 0.01.

A qRT-PCR for apoptosis-related genes showed that the *miR-202-5p* mimic significantly inhibited the expression of BCL2 Apoptosis Regulator (*BCL2*), and increased the expression of Cyclin-Dependent Kinase Inhibitor 1A (*P21*), while the inhibitor significantly increased the expression of *BCL2*, inhibited the expression of *P21* in phGCs (Figure 3A) and hGCs (Figure 3B). Thereafter, the effect of *miR-202-5p* on the apoptosis rate of phGCs and hGCs was further detected by the flow cytometer and observed that the overexpression of miR-202-2p significantly increased the apoptosis rate of phGCs and hGCs (*p* < 0.01), while, when expression of *miR-202-5p* has interfered, the apoptosis rate of both the phGCs and hGCs was significantly decreased (*p* < 0.01) (Figure 3C,D). These results indicated the catalytic role of *miR-202-5p* in the apoptosis of phGCs and hGCs. 

### 2.3. Effect of miR-202-5p on the mRNA Expression Pattern of phGCs

The RNA-seq of phGCs and hGCs transfected with mimics or inhibitors was performed to analyze changes in mRNA expression patterns. An overall number of 631,701,620 clean reads were obtained from 24 samples, with Q30 above 95% and GC content around 50% (Supplementary Table S1). Furthermore, the filtered Clean reads were aligned with the reference genome using HISAT2, as shown in Supplementary Table S2; the lowest mapping rate of 24 samples is 72.02%, and the highest is 76.11% (Supplementary Table S2). These results indicate that the sequencing quality is qualified for further analysis. 

**Figure 4:**
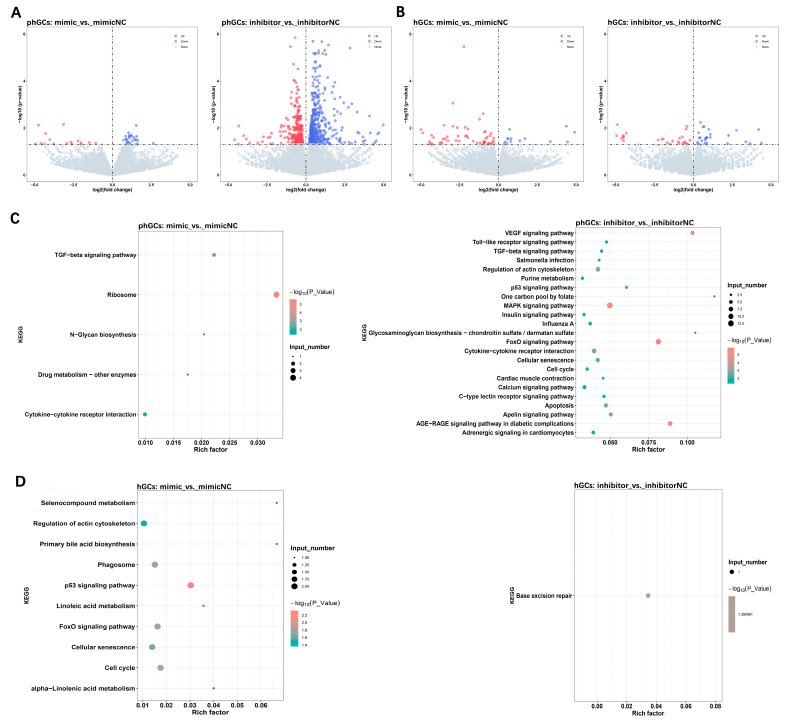
The effect of *miR-202-5p* on mRNA expression profiles of phGCs and hGCs. Volcano plot of DEGs identified in phGCs (**A**) and hGCs (**B**); the significantly enriched KEGG pathways of DEGs identified in phGCs (**C**) and hGCs (**D**).

Based on a screening condition of *p*-value < 0.05, |log2fold change| > 0, in phGCs, 45 DEGs were identified between mimic and mimic-NC, of which 29 were upregulated and 16 were downregulated; a total of 721 DEGs were identified between inhibitor and inhibitor-NC, of which 467 were up-regulated and 253 were down-regulated (Figure 4A). In hGCs, 70 DEGs were identified between mimic and mimic-NC, of which 14 were upregulated and 55 were downregulated; a total of 53 DEGs were identified between inhibitor and inhibitor-NC, of which 27 were up-regulated and 26 were down-regulated (Figure 4B). 

The function enrichment analysis of the obtained DEGs showed that, in phGCs, the DEGs between mimic and mimic-NC were significantly enriched in 112 GO terms; the DEGs between inhibitor and inhibitor-NC were significantly enriched in 56 GO terms. However, in hGCs, DEGs between mimic and mimic-NC are not significantly enriched in GO terms, and DEGs between inhibitor and inhibitor-NC are significantly enriched in positive regulation of gene expression GO terms (Supplementary Figure S1). In addition, in phGCs, the DEGs between mimic and mimic-NC were significantly enriched in 5 KEGG pathways (Figure 4C); the DEGs between inhibitor and inhibitor-NC were significantly enriched in 30 KEGG pathways (Figure 4D). In hGCs, the DEGs between mimic and mimic-NC were significantly enriched in 10 KEGG pathways (Figure 4E); the DEGs between inhibitor and inhibitor-NC were significantly enriched in one KEGG (Figure 4F). 

After joint analysis of all the pathways enriched by DEGs in phGCs, it was found that the majority of significantly enriched pathways were all PI3K/AKT/*FOXO1-*related (Supplementary Table S4). Furthermore, among the DEGs obtained by hGCs, Non-SMC Condensin I Complex Subunit G (NCAPG), Chimerin 1(ARHGAP2), and Meiosis Specific Nuclear Structural 1(MNS1) have also been reported to regulate cell proliferation and apoptosis through PI3K/AKT signal pathway. This result suggests that PI3K/AKT/*FOXO1* signal may be the key pathway for *miR-202-5p* to regulate the proliferation and apoptosis of GCs during follicular selection.

### 2.4. BTBD10 Promotes GCs Proliferation and Suppresses Its Apoptosis

**Figure 5:**
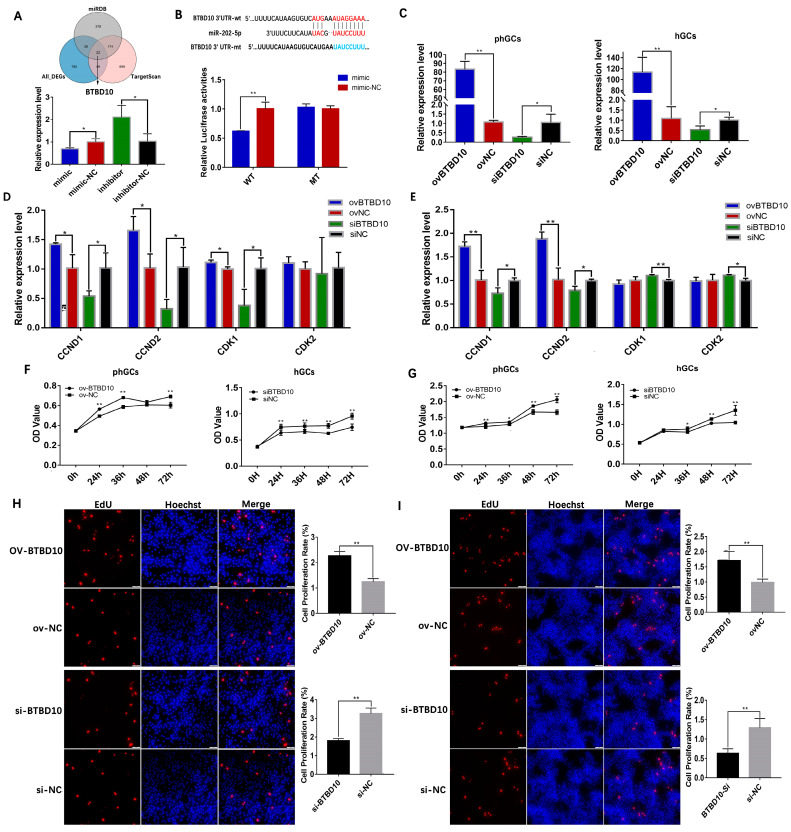
Effect of *BTBD10* on the proliferation of phGCs and hGCs. (**A**): the overlapping genes between TargetScan and miRDB databases predict target genes and DEGs (up), and the validation of the influence of *miR-202-5p* on the expression of predicted genes by qRT-PCR (n = 3); (**B**): schematic illustrating the design of luciferase reporters with *miR-202-5p* binding sites in wild type *BTBD10* 3′ UTR (WT) or mutant type *BTBD10* 3′ UTR (MT) (up), and differences in relative luciferase of *BTBD10* WT and MT vectors after transfection with mimic activities (down) (n = 3); (**C**): identification of overexpression and interference effect of ovBTBD10 and siBTBD10 in phGCs and hGCs (n = 3); the effect of overexpression/interference of *BTBD10* on the expression of proliferation-related genes in phGCs (**D**) and hGCs (**E**) detected by qRT-PCR (n = 3); the effect of overexpression/interference of *BTBD10* on the cell activity of phGCs (**F**) and hGCs (**G**) detected by CCK8 (n = 6); the effect of overexpression/interference of *BTBD10* on the proliferation rate of phGCs (**H**) and hGCs (**I**) detected by EdU staining (n = 3). Data are means ± SD of technical replicates. * *p* < 0.05, ** *p* < 0.01.

The DEGs obtained by transcriptome sequencing were combined with the target genes predicted by TargetScan and miRDB databases to screen the transcriptional target gene of *miR-202-5p*, and 22 predicted target genes were obtained. According to the functional annotation of these 22 predicted target genes, 4 key target genes had been reported to be involved in regulating cell proliferation and apoptosis: Transforming Growth Factor Beta Receptor 1 (*TGFBR1*), SPT6 Homolog, Histone Chaperone And Transcription Elongation Factor (*SUPT6H*), CCR4-NOT Transcription Complex Subunit 6 Like (*CONT6L*), and BTB Domain Containing 10 (*BTBD10*) (Supplementary Table S3). Then, the effect of *miR-202-5p* on the expression level of predicted target genes was further verified by qRT-PCR and demonstrated that the *TGFBR1*, *SUPT6H*, and *BTBD10* were more significantly affected by *miR-202-5p* (Figure 5A, Supplementary Figure S2A). Thereafter, after designing and screening the siRNA with the best interference effect (Supplementary Figure S2B), we verified its effect on the expression of *BCL2* and *CCND1* by qRT-PCR; results showed that *BTBD10* had a more obvious effect on the expression of *CCND1* and *BCL2* (Supplementary Figure S2C), suggesting that *BTBD10* might be a more critical target gene for *miR-202-5p* to regulate the proliferation and apoptosis of geese GCs. Subsequently, the targeting relationship between *miR-202-5p* and *BTBD10* was verified by a dual luciferase assay. As shown in Figure 5B, *miR-202-5p* mimic significantly inhibited the relative fluorescence activity of *BTBD10* wild-type dual luciferase vector (*p* < 0.05), while not affecting the relative fluorescence activity of *BTBD10* binding site mutant dual luciferase vector (*p* > 0.05), indicating that goose *miR-202-5p* can target *BTBD10* by binding to its 3′UTR. These results announced that *BTBD10* is a key target gene for *miR-202-5p* to regulate the proliferation and apoptosis of GCs.

**Figure 6:**
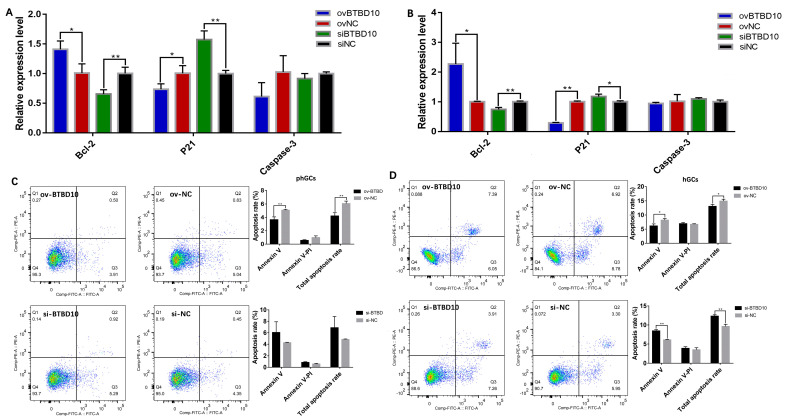
Effect of *BTBD10* on apoptosis of phGCs and hGCs. The relative expression of the apoptosis-related gene in phGCs (**A**) and hGCs (**B**) after overexpression/interference of *BTBD10* (n = 3). The apoptosis rate of phGCs (**C**) and hGCs (**D**) after overexpression/interference of *BTBD10* (n = 3). Data are means ± SD of technical replicates. * *p* < 0.05, ** *p* < 0.01.

The validation results of qRT-PCR for the *BTBD10* overexpression plasmid (ovBTBD10) and small interfering RNA (siRNA) showed that ovBTBD10 significantly increased the expression of *BTBD10*, whereas siBTBD10 significantly decreased the expression of *BTBD10* in both phGCs and hGCs (Figure 5C). A significant increase in *CCND1*, *CCND2* relative expression was observed in pCDNA3.1-*BTBD10* (ovBTBD10) transfected phGCs and hGCs, while a significant decrease was observed in *BTBD10*-siRNA (siBTBD10) transfected phGCs and hGCs (Figure 5D,E). Cell activity analysis further demonstrated that the cell activity of phGCs and hGCs during 24 h–72 h was significantly decreased by the interference of *BTBD10*, while significantly promoted by *BTBD10* overexpression (Figure 5F,G). Likewise, the result of the EdU staining assay showed that the knockdown of *BTBD10* drastically decreased the proliferative rate of phGCs and hGCs (*p* < 0.05), and the overexpression of *BTBD10* significantly increased the proliferative rate of phGCs and hGCs (Figure 6H,I). It was thus demonstrated that *BTBD10* plays a positive role in regulating the proliferation of geese phGCs and hGCs. 

To further explore the role of *miR-202-5p* in GCs apoptosis, the qRT-PCR of apoptosis-related genes and flow cytometer was employed to analyze the changes in cell apoptosis. As shown in Figure 6, after the overexpression of *BTBD10*, the expression of the *BCL2* significantly increased, while the expression of *P21* significantly decreased in phGCs and hGCs. In contrast, the expression of the *BCL2* was significantly decreased, and *P21* was significantly increased by the overexpression of *BTBD10* in phGCs (Figure 6A) and hGCs (Figure 6B). Accordingly, the detection of cell apoptosis rate also revealed that the apoptosis rate of phGCs (Figure 6C) and hGCs (Figure 6D) also significantly decreased, while it significantly increased by the interference of *BTBD10*. It was thus suggested that *BTBD10* plays a negative regulatory role in the apoptosis of geese phGCs and hGCs.

### 2.5. MiR-202-5p Regulates GCs Proliferation and Apoptosis by Targeting BTBD10

**Figure 7:**
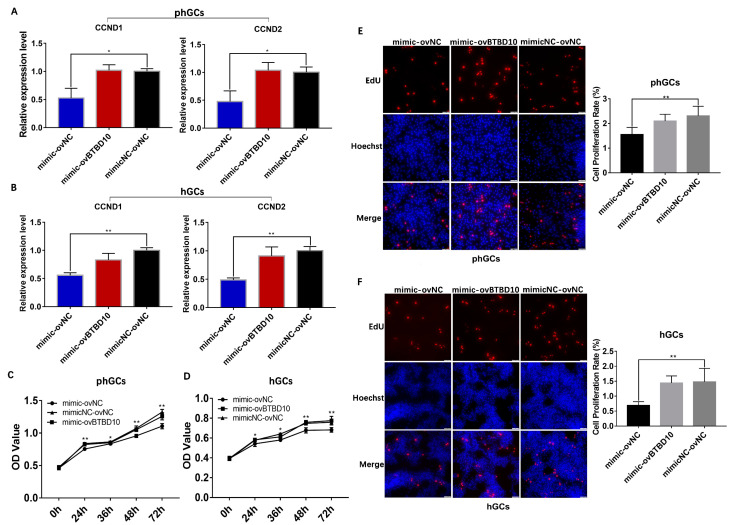
The influence of the co–transfection of *miR-202-5p* mimic and ovBTBD10 on the proliferation of phGCs and hGCs. The co–transfection of mimic and ovBTBD10 on the expression of *CCND1* and *CCND2* in phGCs (**A**) and hGCs (**B**) (n = 3). The influence of co–transfection of mimic and ovBTBD10 in cell activity of phGCs (**C**) and hGCs (**D**) detected by CCK8 (n = 6). The EdU staining was conducted to verify the effect of co–transfection on the proliferation rate of phGCs (**E**) and hGCs (**F**) (n = 3). Data are means ± SD of at least technical replicates. * *p* < 0.05, ** *p* < 0.01.

A co-transfection assay was performed to investigate the relationship between *miR-202-5p* and *BTBD10* in the proliferation of phGCs and hGCs. The result of qRT-PCR showed that compared to the cells co-transfected with mimic and ovNC, the expression of *CCND1* and *CCND2* in phGCs (Figure 7A) and hGCs (Figure 7B) was restored by co-transfection of mimic and ovBTBD10 to a level that was not significantly different from the control group which co-transfected with mimicNC and ovNC. Similarly, the co-transfection with mimic and ovBTBD10 also significantly restored the negative effect of *miR-202-5p* overexpression on cell activity (Figure 7C,D) and proliferation rate (Figure 7E,F) of phGCs and hGCs.

**Figure 8:**
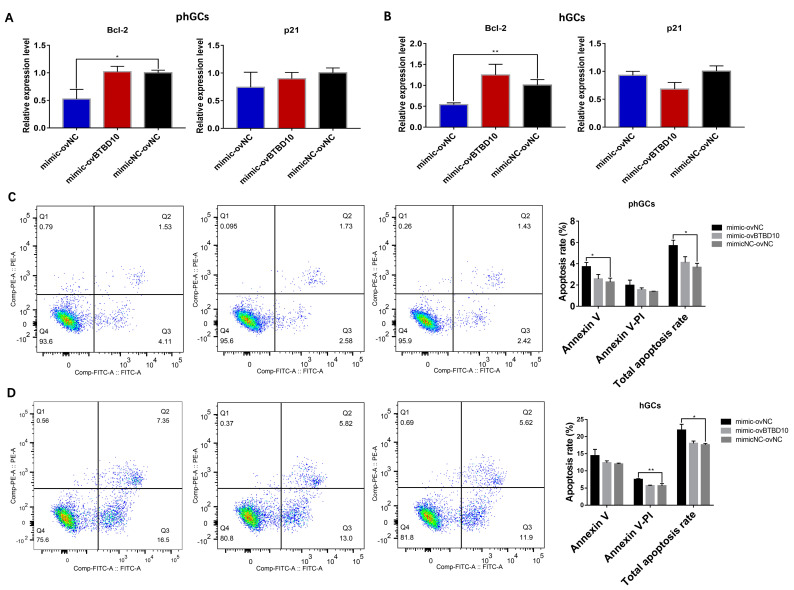
Effect of co–transfection of mimic and ovBTBD10 on phGCs and hGCs apoptosis. The effect of the co–transfection of *miR-202-5p* mimic and ovBTBD10 on the expression of *BCL2* and *P21* in phGCs (**A**) and hGCs (**B**) (n = 3); the effect of co–transfection of *miR-202-5p* mimic and ovBTBD10 on apoptosis rate of phGCs (**C**) and hGCs (**D**) detected by flow cytometer (n = 3). Data are means ± SD of technical replicates. * *p* < 0.05, ** *p* < 0.01.

The expression of apoptosis-related genes also was detected to determine the effect of the co-transfection on GCs apoptosis. Results showed that compared to the mimic and ovNC co-transfection group, the co-transfection of mimic and *BTBD10* significantly increased the expression of the anti-apoptotic gene *BCL2* in phGCs (Figure 8A) and hGCs (Figure 8B). Meanwhile, the apoptosis rate of phGCs (Figure 8C) and hGCs (Figure 8D) detected by the flow cytometer also showed a significant decrease in the group co-transfection with mimic and ovBTBD10. These results demonstrate that *BTBD10* can protect the phGCs and hGCs from *miR-202-5p*-induced apoptosis.

### 2.6. miR-202-5p Regulate BTBD10/PIK3CB/AKT1

**Figure 9:**
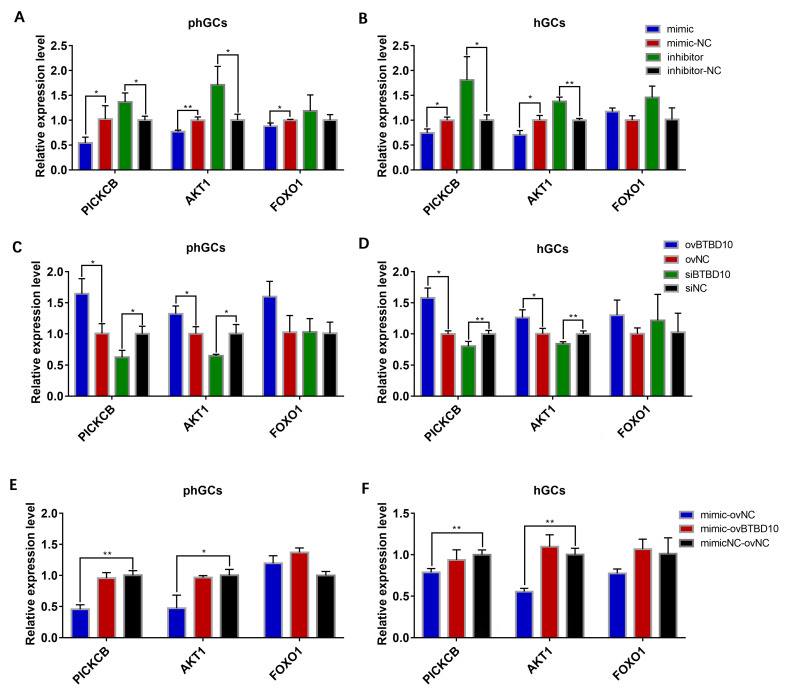
The effect of *miR-202-5p* and *BTBD10* on the expression of *PIK3CB*/*AKT1/FOXO1* pathway. The effect of *miR-202-5p* mimic/inhibitor on the expression of *PIK3CB*/*AKT1/FOXO1* in *ph*GCs (**A**) and hGCs (**B**) (n = 3); the effects of ovBTBD10 and siBTBD0 on the expression of *PIK3CB*/*AKT1/FOXO1* in *ph*GCs (**C**) and hGCs (**D**) (n = 3); the effect of *miR-202-5p* mimic and ovBTBD10 co-transfection on *PIK3CB*/*AKT1/FOXO1* expression in phGCs (**E**) and hGCs (**F**) (n = 3). Data are means ± SD of technical replicates. * *p* < 0.05, ** *p* < 0.01.

According to the transcriptome sequence results, the *PIK3CB*/*AKT1/FOXO1 signa*l pathway may be the key pathway for *miR-202-5p* to regulate the proliferation and apoptosis of goose GCs. Therefore, the effect of *miR-202-5p* overexpression/interference, *BTBD10* overexpression/interference, and co-transfection of mimic and ovBTBD10 on the expression of *PIK3CB*, AKT Serine/Threonine Kinase 1 (*AKT1)*, and Forkhead Box O1 (*FOXO1)* was further verified by qRT-PCR. As shown in Figure 9, the overexpression of *miR-202-5p* significantly inhibited the expression of Phosphatidylinositol-4,5-Bisphosphate 3-Kinase Catalytic Subunit Beta (*PIK3CB*) and *AKT1,* while the interference of *miR-202-5p* significantly promoted the expression of *PIK3CB* and *AKT1* in *ph*GCs (Figure 9A) and hGCs (Figure 9B). On the contrary, the overexpression of *BTBD10* significantly increased the expression of *PIK3CB* and *AKT1*, and the interference of *BTBD10* significantly decreased the expression of *PIK3CB* and *AKT1* in *ph*GCs (Figure 9C) and hGCs (Figure 9D). Notably, the co-transfection of mimic and ovBTBD10 significantly restored the inhibitory effect of *miR-202-5p* mimic on the expression of PI3K, and *AKT1* in *ph*GCs (Figure 9E) and hGCs (Figure 9F). However, there was no significant effect on the expression of *FOXO1.* It was thus summarized that the *PIK3CB*/*AKT1* signaling pathway is the key signaling pathway for *miR-202-5p* regulating the proliferation and apoptosis of goose GCs by targeting *BTBD10*.

## 3. Discussion

The proliferation and apoptosis of granulosa cells play a critical role in the follicular selection, which is closely related to egg production performance. Therefore, it is of great value to fully clarify the regulation mechanism of granulosa cell proliferation and apoptosis during follicular selection. During follicular development, such as primordial follicular development, and follicular atresia, a large number of differentially expressed miRNAs have been identified by microarray analysis of miRNAs [22]. This suggests that miRNA is an important regulator of follicular development. *miR-202-5p* is a cancer miRNA that inhibits the proliferation and promotes apoptosis of ovarian cancer cells [23], breast cancer cells [24], pancreatic cancer cells [25], etc. Our findings indicate that *miR-202-5p* inhibits cell proliferation and promotes apoptosis in phGCs and hGCs during goose follicle selection. This is consistent with previous research demonstrating that *miR-202-5p* targets TGFBR2 to promote apoptosis and inhibit proliferation in goat GCs [26]. The detection results of *miR-202-5p* expression patterns in follicular granulosa cells at different stages showed that compared to the phGCs, the expression of *miR-202-5p* in hGCs was obviously downward trend (although not significantly), consistent with the sequencing results of Lin et al. [15]. The ovarian follicle selection is a complex and finely tuned process that entails a series of signal cascades involving both activation and suppression [27]. For instance, before follicle selection, GC differentiation is suppressed by active mitogen-activated protein kinase (MAPK) signaling via extracellular signal-regulated kinases (*ERK1*, *ERK2*), and the process of follicular selection is accompanied by the inhibition of *MAPK/ERK1/ERK2* signaling [1]. Therefore, we hypothesize that, along with the progression of follicular selection, the *miR-202-5p* expression will be inhibited to relieve its negative impact on follicular selection. It was also observed that the expression of *miR-202-5p* in the ovary of single lambs goats was significantly higher than that of multiple lambs goats [28]. In addition, Wang et al. conducted miRNA-Seq on the whole ovarian tissue of hens not yet laying at 60 days, 100 days, 140 days, and at 140 days for laying hens, and found that the expression level of *miR-202-5p* in the ovaries of the laying hens (140 days old) was significantly lower than that of any other group [29]. These reports further support the hypothesis that the expression of *miR-202-5p* was negatively correlated with follicular selection. In summary, *miR-202-5p* may be an important negative regulator of goose follicular selection. The inhibition of *miR-202-5p* expression is believed to be a critical step in promoting follicular selection. These findings provide valuable insights for researchers seeking to deepen their understanding of the regulation of goose follicular selection. Our further research will focus on the upstream molecular mechanisms that regulate *miR-202-5p*, such as the effects of FSH and LH on the expression and function of *miR-202-5p*. The miRNAs promote the degrading and inhibiting translation of mRNA by interacting with their 5′ and 3′ untranslated region (UTR) sequences. Our results showed that *TGFBR1* [30], *SUPT6H* [31], *CNOT6L* [32], and *BTBD10* [33], which were reported to participate in the regulation of cell apoptosis and follicular development, may be the key target genes for *miR-202-5p* to regulate goose GCs. Subsequently, the result of qRT-PCR indicates that *miR-202-5p* impacted the expression of *BTBD10* most significantly. It suggests that *BTBD10* is the key target gene for *miR-202-5p* to regulate the proliferation and apoptosis of geese GCs. Thereafter, we identified that *BTBD10* acts as an important regulator for promoting GCs proliferation and inhibiting GCs apoptosis, and mediates *miR-202-5p* to inhibit GC proliferation and promote apoptosis during follicular selection. *BTBD10* is a protein 10 containing the BTB/POZ domain. It is reported that the BTB/POZ domain can inhibit gene transcription by interacting with the components of the histone deacetylase co-repressor complex. In addition, BTB/POZ domain also has been revealed to be related to cytoskeletal tissue development and carcinogenesis [34,35]. Taken together, the *BTBD10*-mediated regulation of *miR-202-5p* on the proliferation and apoptosis of GCs may be the key molecular mechanism for geese follicular selection.

Some studies have indicated that overexpression of *BTBD10* leads to an increase in the phosphorylation levels of AKT, while a decrease in the endogenous level of *BTBD10* results in a reduction in AKT phosphorylation levels [36]. In vitro analysis has demonstrated that *BTBD10* interacts with protein phosphatase 2A (PP2A) and inhibits the dephosphorylation of AKTs by PP2A, which indicates that *BTBD10* can interact with PP2A to maintain AKT phosphorylation [36]. These results show that *BTBD10* is an important AKT activator, which is an inhibitor of cell death by activating AKT [36,37]. Meanwhile, the results of transcriptome sequencing also showed that almost all the pathways enriched by DEGs were *PIK3CB*/*AKT1-*related, this further supports the hypothesis that PI3K/AKT signal is the key pathway for *miR-202-5p* to regulate the proliferation and apoptosis of goose GCs. Therefore, the effect of *miR-202-5p*, *BTBD10*, and their co-transfection on the expression of *PIK3CB* and *AKT1* was tested, and found that the expression of *PIK3CB* and *AKT1* is negatively regulated by *miR-205-2p*, but restored by *BTBD10*. One of the subunits of PI3K is encoded by *PIK3CB*. The *AKT1* is a central mediator of PI3K pathway and an important downstream target of the PI3K. PIKCB/*AKT1* signaling is the crucial pathway controlling cell growth and metabolism [38,39]. This suggested that *miR-202-5p* may regulate the proliferation and apoptosis of geese phGCs and hGCs by targeting *BTBD10* to suppress the activity of *PIK3CB*/*AKT1* signaling. 

## 4. Materials and Methods

### 4.1. Animals

The healthy maternal line of Tianfu meat geese (*Anser cygnoides*) at the peak of egg production was used as study participants. These geese are raised in the Waterfowl Breeding Experimental Farm at Sichuan Agricultural University (Sichuan, China) under the conditions of natural light, temperature conditions, as well as unlimited food and water. According to the egg-laying records, three geese, at the age of 35–45 weeks and laying in regular sequences of at least 2–3 eggs, and after being determined by the worker touching the abdomen that there are hard shell eggs in the fallopian tube, were used to collect the follicles for each experiment. Based on their diameter, the ovarian follicles were divided into two classes: pre-hierarchical (2 to 4 mm, 6 to 8 mm, 8 to 10 mm in diameter) and hierarchical (from F5–F1, F1 > F2 > F3 > F4 > F5). 

### 4.2. GC Culture and Transfection

Previously, granulosa layers are separated from theca layers in each follicle [40]. Firstly, after isolating the granulosa layers of follicles, the granulosa layers of 10–20 pre-hierarchical follicles (typically measure between 8 and 10 mm in diameter using a vernier caliper) and 3 hierarchical follicles (F2–F4 large yellow follicles) are mixed and carefully sectioned into small pieces within two sterile 5 mL centrifuge tubes, respectively. Subsequently, PBS (pH 7.3) was used to wash the granulosa layers, followed by the digestion of type II collagenases (Sigma, Aldrich, St. Louis, MI, USA) at a concentration of 0.1%. After dilution to 5 × 10^5^ cells/mL in Dulbecco’s Modified Eagle’s Medium/Nutrient Mixture (F12) with 3% fetal bovine serum, the cells were cultured on 12-well culture plates for the RNA sample and apoptosis rate detection sample or 96-well culture plates for CCK8 and EdU staining at 37 °C in a humidified atmosphere of 5% CO_2_ and 95% air. A fresh medium was used for GC culture before DNA or RNA transfection. Then, according to the manufacturer’s instructions, Lipofectamine 3000 (ThermoFisher Scientific, Carlsbad, CA, USA) was used to transfect mimic (UUUCCUAUGCAUAUACUUAUUUU), mimic-NC (UUGUACUACACAAAAGUACUG), inhibitor (AAAGAAGUAUAUGCAUAGGAAA), inhibitor-NC (CAGUACUUUUGUGUAGUACAA), *BTBD10* overexpression plasmid, and siRNAs (Supplementary Table S4). After 24 h of transfection, cells were harvested for RNA extraction.

### 4.3. Total RNA Extraction, Library Preparation, and Sequencing

The TRIzol^®^ Reagent (Plant RNA Purification Reagent for plant tissue) was used to extract the total RNA the according to the manufacturer’s instructions (Invitrogen, Waltham, CA, USA) and genomic DNA was removed by DNase I (TaKaRa, Dalian, China). Subsequently, the quality of extracted total RNA was determined by 2100 Bioanalyser (Agilent Technologies, Santa Clara, CA, United States) and the ND-2000 (NanoDrop Technologies, Wilmington, DE, USA) was employed to quantify total RNA. Only the RNA satisfying the OD260/280 = 1.8~2.2, OD260/230 ≥ 2.0, RIN ≥ 6.5, 28S:18S ≥ 1.0, >1 μg can was used to construct the sequencing library.

The TruSeqTM RNA sample preparation kit was used to prepare the RNA-seq transcriptome library. Then, the paired-end RNA-seq sequencing library quantized by TBS380 was sequenced with the Illumina HiSeq X ten/NovaSeq 6000 sequencer (2 × 150 bp read length). All obtained RNA data in the current study are available from the BioProject database (PRJNA821376). The mapped reads of each sample were assembled by StringTie (v1.3.3) [41] using a reference-based approach. Differentially expressed genes (DEGs) were counted using the DESeq2 package of R. For biological replicates, *p*-value < 0.05, and |Fold change| > 0 analyzed by EBseq were considered to indicate significant differences in mRNA expressions. Gene Ontology (GO) and Kyoto Encyclopedia of Genes and Genomes (KEGG) enrichment analyses were conducted by KOBAS 3.0 online2 [42].

### 4.4. Quantitative Real-Time PCR

Following the TRIzol reagent (Invitrogen, Carlsbad, CA, United States) extraction of total RNA, spectrophotometric absorbance measurements were conducted to determine the quality, purity, and concentration of RNA. According to the manufacturer’s instructions, the cDNA was further synthesized from total RNA using a PrimeScript RTTM Reagent Kit (TaKaRa, Dalian, China). The 2 × SYBR Premix Ex Taq II (TaKaRa, Dalian, China) was employed to conduct the qRT-PCR. Using 1 μL cDNA, 6.25 μL of SYBR Ex Taq, 4.25 μL of ddH_2_O, and 0.5 μL of each gene-specific primer (10 μM), a total of 12.5 μL reaction solution was prepared. For each sample, the Ct value of each gene were normalized to GAPDH using the 2^–ΔΔCt^ method [43] and significance analyses were repeated three times through t-test, one–way ANOVA, and non-parametric test. The primers for qRT–PCR are summarized in Table 1. 

### 4.5. Annexin V-FITC/PI Double Staining in the Detection of Apoptosis by Flow Cytometry

After digestion with 0.25% trypsin-EDTA, the cell suspension was stained by the Annexin V/PI cell apoptosis detection kit (Beyotime Biotech, Nantong, China) according to the manufacturer’s instructions to detect the changes in cell apoptosis. BD Accuri C6 Flow cytometers were used to quantify apoptotic cells, and FlowJo software (v 10.8.1) was used to analyze the data; then the sum of early and late apoptosis subpopulations was calculated as the apoptosis rate [44].

### 4.6. Dual Luciferase Assays

The predicted target genes for goose *miR-202-5p* regulating GCs proliferation and apoptosis were analyzed by using both miRDB (http://mirdb.org/ accessed on 19 January 2022) and TargetScan (https://www.targetscan.org/vert_80/ accessed on 19 January 2022) and its crosstalk with DEGs. Then, the HEK 293T cells in 48-well plates co-transfected with plasmids (wild-type or mutant pmiRGLO-3′UTR-*BTBD10*) (2 μg) and *miR-202-5p* mimics or mimic-NC (2 μL) were lysed to measure the luciferase activities using a dual-luciferase reporter assay system (Beyotime, Shanghai, China). A normalization of firefly activity to renilla activity was used to determine the relative luciferase activities [45].

### 4.7. 5-Ethynyl-2′-Deoxyuridine (EdU) Assay

We performed GC proliferation assays using the using Cell-Light^TM^ EdU Apollo Vitro Kit (RiboBio, Guangzhou, China). According to the formula below, the EdU incorporation rate was calculated as the ratio of the number of EdU-incorporated cells to the number of Hoechst 33342-staining cells. At least 500 cells were counted for every group.

### 4.8. Cell-Counting Kit-8 Assay (CCK-8)

The cell activity in GCs was tested by Cell Counting Kit-8 assay. Briefly, CCK-8 solution (MeilunBio, Dalian, China) (10 μL/well) was added to transfected cells planted in 96-well plates (6 × 10^3^ /well). Then, after staining for 4 h at 37 °C, the optical density (OD) value (450 nm) was evaluated by spectrophotometry.

### 4.9. Statistical Analysis

Experiment data for each group were collected from a minimum of three individual geese at the same time, and each experimental procedure was set up with at least three technical repetitions, data are the means ± SEM. Then, the obtained data underwent initial analysis for normal distribution using descriptive statistics in SPSS, followed by significance analysis through t-tests, one-way ANOVA, and non-parametric tests using SPSS (version 20.0, IBM, Armonk, IL, USA), those with *p*-value < 0.05 were considered as significant and *p*-value < 0.01 were considered as extremely significant differences.

## 5. Conclusions

**Figure 10:**
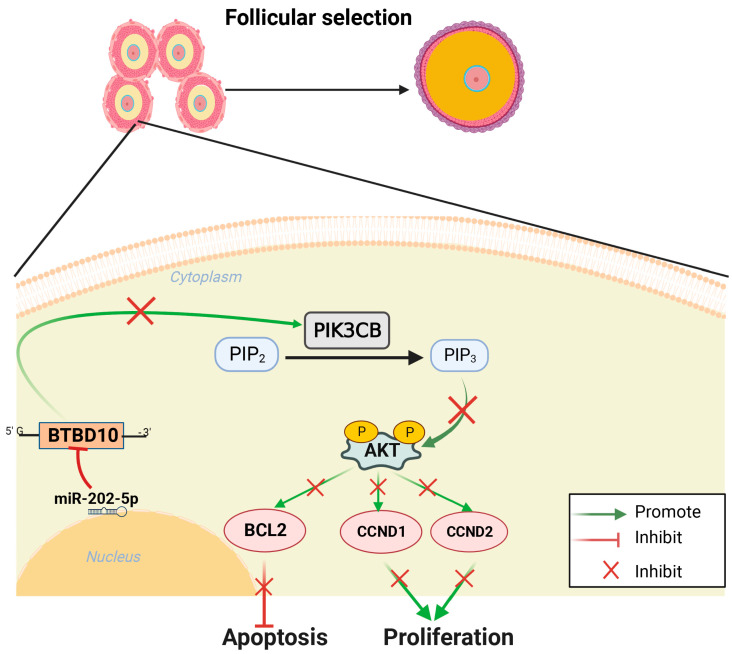
Molecular mechanism prediction of *miR-202-5p* regulating proliferation and apoptosis of GCs during the geese follicular selection.

In conclusion, we observed that *miR-202-5p* was highly expressed in GCs compared with TCs in follicles at different developmental stages, and confirmed that *miR-202-5p* could inhibit the proliferation of phGCs and hGCs, and promote their apoptosis. Furthermore, we established that *miR-202-5p* suppresses the proliferation and promotes apoptosis of phGCs and hGCs by targeting *BTBD10* to keep down the activity of *PIK3CB*/*AKT1* signaling (Figure 10). Therefore, the results obtained in this study revealed that *miR-202-5p* is a negative regulator of goose follicular selection. These findings are expected to provide valuable theoretical references for understanding the molecular mechanism of goose follicular selection, as well as provide a potential target for goose molecular-assistance breeding to improve annual egg production. 

## Figures and Tables

**Table 1:** Primer pairs for real-time quantitative PCR.

Gene	Forward Primer (5′–3′)	Reversed Primer (5′–3′)	Tm (°C)	Product Length (bp)
***CCND1***	TTCATCGCCCTTTGTGCC	ATTGCTCCCACGCTTCCA	60	80
***PCNA***	TGTTCCTCTGGTTGTGGAGTA	GAGCCTTCTTGTTGGTCTTCA	58	90
***CCND2***	CCACCGTCAATGATAGCAACT	AGGAAGTCTGTTAGGCTGTCA	58	104
***CDK1***	GCAAGGTATCGTCTTCTGTCAT	CCAATCCAAAGTCTGCCAGTT	58	110
***CDK2***	CTCCACCTCCAAGTTCCTAATG	GCTGATCTATGGCACTGTCC	58	89
***P21***	TGAGGCAACACCTGGAAGAAG	CCTTAGATGGGACCTTGTGGG	60	207
***BCL2***	GATGCCTTCGTGGAGTTGTATG	GCTCCCACCAGAACCAAAC	60	100
***CAS-3***	CTGGTATTGAGGCAGACAGTGG	CAGCACCCTACACAGAGACTGAA	62	158
***FAS***	CCAGCAGAACCCAGGTGAAA	GGGAGTGTCATCTCTTCCGC	57	108
**GAPDH**	TTTCCCCACAGCCTTAGCA	GCCATCACAGCCACACAGA	60	90
***miR-202-5p***	GCGCTTTCCTATGCATATACT	CAGGTCCAGTTTTTTTTTTTTTT	55	
**U6**	TACAGAGAAGATTAGCATGG	CAGGTCCAGTTTTTTTTTTTTTT	55	

## Data Availability

Not applicable.

## Supplementary Materials

The following supporting information can be downloaded at: https://www.mdpi.com/article/10.3390/ijms24076792/s1.

## Abbreviations

GCs, granulosa cells; TCs, theca cell; phGCs, pre-hierarchical follicles; hGCs, hierarchical follicles; DEGs, differentially expressed genes; *BTBD10*, BTB Domain Containing 10; *PIK3CB*, Phosphatidylinositol-4,5-Bisphosphate 3-Kinase Catalytic Subunit Beta; *AKT1, AKT* Serine/Threonine Kinase 1; TGFBR1, Transforming Growth Factor Beta Receptor 1; SUPT6H, SPT6 Homolog, Histone Chaperone And Transcription Elongation Factor; CNOT6L, CCR4-NOT Transcription Complex Subunit 6 Like; *CCND1*, Cyclin D1; *CCND2*, Cyclin D2; *CDK1*, Cyclin Dependent Kinase 1; *CDK2*, Cyclin Dependent Kinase 2; *PCNA*, Proliferating Cell Nuclear Antigen; *BCL2*, BCL2 Apoptosis Regulator; *P21*, Cyclin Dependent Kinase Inhibitor 1A; *CAS-3*, Caspase 3; *FAS*, Fas Cell Surface Death Receptor; NCAPG, Non-SMC Condensin I Complex Subunit G; ARHGAP2, Chimerin 1; MNS1, Meiosis Specific Nuclear Structural 1; *FOXO1, Fork*head Box O1; ovBTBD10, *BTBD10* overexpression vector; siBTBD10, *BTBD10* siRNA
