# Supplementary material for: MiR-202-5p Regulates Geese Follicular Selection by Targeting BTBD10 to Regulate Granulosa Cell Proliferation and Apoptosis
Source: Int J Mol Sci. 2023 Apr 5;24(7):6792. doi: 10.3390/ijms24076792 (PMC10095183; doi:10.3390/ijms24076792)
Supplement: Supplementary file 1 [file ijms-24-06792-s001.zip › Supplementary Table S1-Overview of RNA sequencing data.pdf]

Table S1. Overview of sequencing data

Sample_ID	Clean reads	clean bases	Q20%	Q30%	GC%
IN_8_1	28795713	8.55E+09	98.52	95.47	49.27
IN_8_2	27256910	8.09E+09	98.59	95.65	49.04
IN_8_3	25850930	7.66E+09	98.59	95.66	49.39
IN_F_1	26780573	7.97E+09	98.56	95.52	48.82
IN_F_2	25827356	7.65E+09	98.48	95.35	49.41
IN_F_3	25774002	7.66E+09	98.51	95.4	48.9
IN_NC_8_1	24890385	7.4E+09	98.52	95.42	49.19
IN_NC_8_2	26408756	7.85E+09	98.55	95.53	49.21
IN_NC_8_3	26600981	7.92E+09	98.54	95.49	48.59
IN_NC_F_1	27088626	8.05E+09	98.56	95.55	48.98
IN_NC_F_2	25508839	7.52E+09	98.57	95.61	49.53
IN_NC_F_3	26808815	7.99E+09	98.49	95.31	48.05
MI_8_1	23024858	6.85E+09	98.54	95.48	49.17
MI_8_2	27466894	8.07E+09	98.48	95.42	50.06
MI_8_3	28757092	8.51E+09	98.55	95.54	48.9
MI_F_1	27680745	8.22E+09	98.57	95.55	48.62
MI_F_2	24435025	7.25E+09	98.42	95.17	48.53
MI_F_3	29767880	8.75E+09	98.59	95.68	49.54
MI_NC_8_1	25780268	7.73E+09	98.54	95.5	49.66
MI_NC_8_2	27673077	8.22E+09	98.59	95.63	49.28
MI_NC_8_3	24181221	7.21E+09	98.56	95.57	48.84
MI_NC_F_1	25389159	7.57E+09	98.57	95.54	49
MI_NC_F_2	26374863	7.83E+09	98.57	95.58	48.68
MI_NC_F_3	23578652	7.03E+09	98.46	95.23	48.88
