# Supplementary material for: MiR-202-5p Regulates Geese Follicular Selection by Targeting BTBD10 to Regulate Granulosa Cell Proliferation and Apoptosis
Source: Int J Mol Sci. 2023 Apr 5;24(7):6792. doi: 10.3390/ijms24076792 (PMC10095183; doi:10.3390/ijms24076792)
Supplement: Supplementary file 1 [file ijms-24-06792-s001.zip › Supplementary Table S2 Mapping rate of RNA sequence results.pdf]

Table S2. Mapping rate of sequence results

Sample ID	Total mapped	Multiple mapped	Uniquely mapped
IN_8_1	19909245(74.34%)	557546(2.08%)	19351699(72.26%)
IN_8_2	19068945(73.83%)	512362(1.98%)	18556583(71.85%)
IN_8_3	19493817(75.63%)	546699(2.12%)	18947118(73.51%)
IN_F_1	21243030(73.77%)	780992(2.71%)	20462038(71.06%)
IN_F_2	20237625(74.25%)	692979(2.54%)	19544646(71.71%)
IN_F_3	19116216(73.95%)	663461(2.57%)	18452755(71.38%)
IN_NC_8_1	18694502(75.11%)	726184(2.92%)	17968318(72.19%)
IN_NC_8_2	19809792(75.01%)	772420(2.92%)	19037372(72.09%)
IN_NC_8_3	20014442(75.24%)	736954(2.77%)	19277488(72.47%)
IN_NC_F_1	20085102(74.15%)	539247(1.99%)	19545855(72.16%)
IN_NC_F_2	18776461(73.61%)	455812(1.79%)	18320649(71.82%)
IN_NC_F_3	20406243(76.11%)	534749(1.99%)	19871494(74.12%)
MI_8_1	17282645(75.06%)	681826(2.96%)	16600819(72.1%)
MI_8_2	19901676(72.45%)	509076(1.85%)	19392600(70.6%)
MI_8_3	21755848(75.65%)	1161779(4.04%)	20594069(71.61%)
MI_F_1	20524698(74.15%)	627171(2.27%)	19897527(71.88%)
MI_F_2	17597151(72.02%)	429161(1.76%)	17167990(70.26%)
MI_F_3	21919442(73.64%)	802321(2.7%)	21117121(70.94%)
MI_NC_8_1	18903834(73.33%)	724711(2.81%)	18179123(70.52%)
MI_NC_8_2	20882426(75.46%)	885750(3.2%)	19996676(72.26%)
MI_NC_8_3	17950801(74.23%)	929725(3.84%)	17021076(70.39%)
MI_NC_F_1	18697835(73.65%)	652377(2.57%)	18045458(71.08%)
MI_NC_F_2	19168135(72.68%)	516699(1.96%)	18651436(70.72%)
MI_NC_F_3	17425964(73.91%)	715734(3.04%)	16710230(70.87%)
