# Supplementary material for: MiR-202-5p Regulates Geese Follicular Selection by Targeting BTBD10 to Regulate Granulosa Cell Proliferation and Apoptosis
Source: Int J Mol Sci. 2023 Apr 5;24(7):6792. doi: 10.3390/ijms24076792 (PMC10095183; doi:10.3390/ijms24076792)
Supplement: Supplementary file 1 [file ijms-24-06792-s001.zip › Supplementary Table S3-siRNA sequence.pdf]

Table S3. siRNA

	sense (5′–3′)	anti-sense (5′–3′)
si-BTBD10-434	GGAGCUCGGAAUGUAAGAATT	UUCUUACAUUCCGAGCUCCTT
si-BTBD10-950	GAGGAGUAUUCACAAAUUATT	UAAUUUGUGAAUACUCCUCTT
si-BTBD10-1122	GGCCAGAGGUGAUUUACAATT	UUGUAAAUCACCUCUGGCCTT
si- SUPT6H-1056	GCACCAUCCAGAAGAUUAATT	UUAAUCUUCUGGAUGGUGCTT
si- SUPT6H-2790	GCAGCUCUGAUGAGGAUAUTT	AUAUCCUCAUCAGAGCUGCTT
si- SUPT6H-4182	GCUCCACGCUUUGGAUUAATT	UUAAUCCAAAGCGUGGAGCTT
si- TGFBR1-542	GAGGCAGAAAUUUAUCAAATT	UUUGAUAAAUUUCUGCCUCTT
si- TGFBR1-860	GACCUAGGAUUGGCAGUUATT	UAACUGCCAAUCCUAGGUCTT
si- TGFBR1-1062	GUGGAAUCCAUGAAGAUUATT	UAAUCUUCAUGGAUUCCACTT
