# Supplementary material for: MiR-202-5p Regulates Geese Follicular Selection by Targeting BTBD10 to Regulate Granulosa Cell Proliferation and Apoptosis
Source: Int J Mol Sci. 2023 Apr 5;24(7):6792. doi: 10.3390/ijms24076792 (PMC10095183; doi:10.3390/ijms24076792)
Supplement: Supplementary file 1 [file ijms-24-06792-s001.zip › Supplementary Table S4 Function annotation of predicted target genes.pdf]

Table S4. Function annotation of predicted target genes

Target Gene	Function
**PPM1E**	Protein phosphatase that inactivates multifunctional CaM kinases such as CAMK4 and CAMK2
**SUPT6H**	Transcription elongation factor which binds histone H3 and plays a key role in the regulation of transcription elongation and mRNA processing
**FGD6**	May activate CDC42, a member of the Ras-like family of Rho- and Rac proteins, by exchanging bound GDP for free GTP.
**MIER1**	Transcriptional repressor regulating the expression of a number of genes including SP1 target genes
**FAM19A2**	Has a role as neurotrophic factor involved in neuronal survival and neurobiological functions.
**GPBP1L1**	Possible transcription factor
**YAF2**	Binds to MYC and inhibits MYC-mediated transactivation
**FBXO9**	directly regulating mTOR signaling
**MKL2**	Acts as a transcriptional coactivator of serum response factor (SRF).
**KDM4A**	playing a central role in histone code
**CNOT6L**	Mediates cell proliferation and cell survival and prevents cellular senescence
**PTCH1**	Acts as a receptor for sonic hedgehog (SHH), indian hedgehog (IHH) and desert hedgehog (DHH)
**TET2**	plays a key role in active DNA demethylation
**BTBD10**	Plays a major role as an activator of AKT family members by inhibiting PPP2CA-mediated dephosphorylation, thereby keeping AKTs activated
**TGFBR1**	Transmembrane serine/threonine kinase forming with the TGF-beta type II serine/threonine kinase receptor
**TGFBR2**	Transmembrane serine/threonine kinase forming with the TGF-beta type II serine/threonine kinase receptor
**FMNL2**	Plays a role in the regulation of cell morphology and cytoskeletal organization
**TFDP2**	Can stimulate E2F-dependent transcription.
**CMPK1**	Catalyzes the phosphorylation of pyrimidine nucleoside monophosphates at the expense of ATP
**SIK1**	Serine/threonine-protein kinase involved in various processes such as cell cycle regulation, gluconeogenesis and lipogenesis regulation
**TLN2**	As a major component of focal adhesion plaques that links integrin to the actin cytoskeleton, may play an important role in cell adhesion
**E2F7**	Atypical E2F transcription factor that participates in various processes such as angiogenesis, polyploidization of specialized cells and DNA damage response.
