# Supplementary material for: MiR-202-5p Regulates Geese Follicular Selection by Targeting BTBD10 to Regulate Granulosa Cell Proliferation and Apoptosis
Source: Int J Mol Sci. 2023 Apr 5;24(7):6792. doi: 10.3390/ijms24076792 (PMC10095183; doi:10.3390/ijms24076792)
Supplement: Supplementary file 1 [file ijms-24-06792-s001.zip › Suppmentary-Figure S1 GO enrichment analysis of DEGs.pdf]

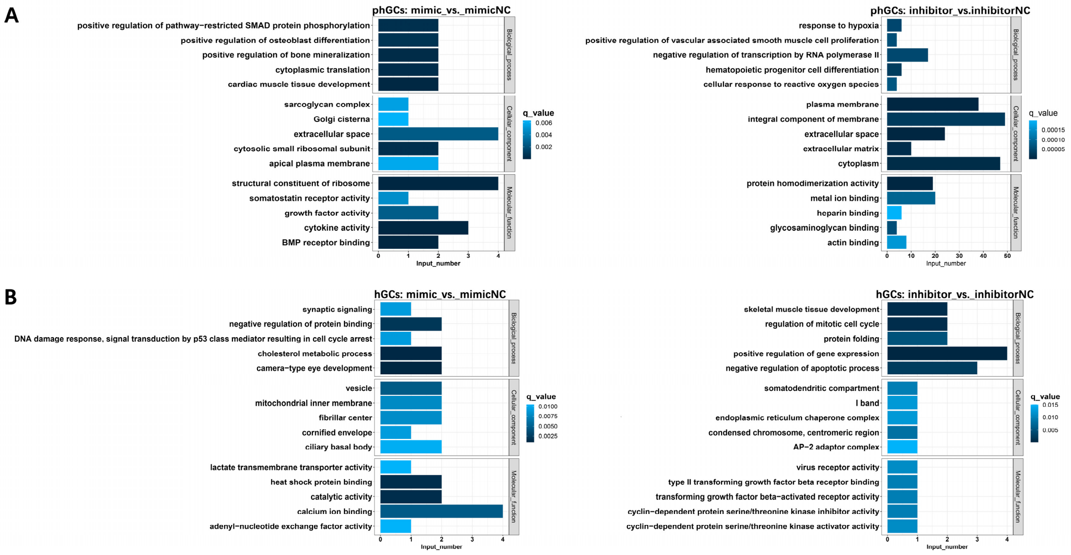

Figure S1. GO enrichment analysis of DEGs. The top five most significant GO terms enriched by DEGs identified in phGCs (A) and hGCs (B) under the cellular_component, biological_process, and molecular_function classification, respectively.
