# Supplementary figures and images for: MiR-202-5p Regulates Geese Follicular Selection by Targeting BTBD10 to Regulate Granulosa Cell Proliferation and Apoptosis
Source: Int J Mol Sci. 2023 Apr 5;24(7):6792. doi: 10.3390/ijms24076792 (PMC10095183; doi:10.3390/ijms24076792)

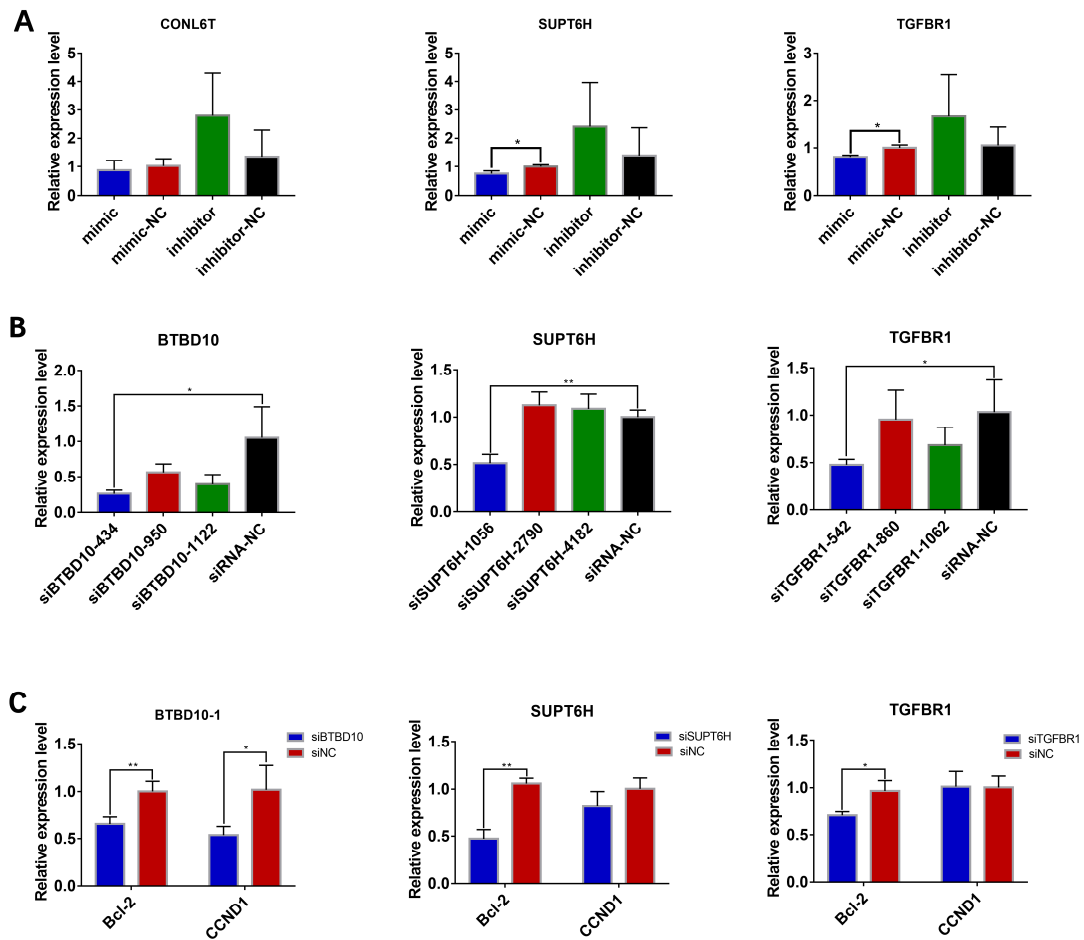

Supplement: Supplementary file 1 [file ijms-24-06792-s001.zip › Suppmentary-Figure S2 Identification of miR-202-5p target gene.pdf]
